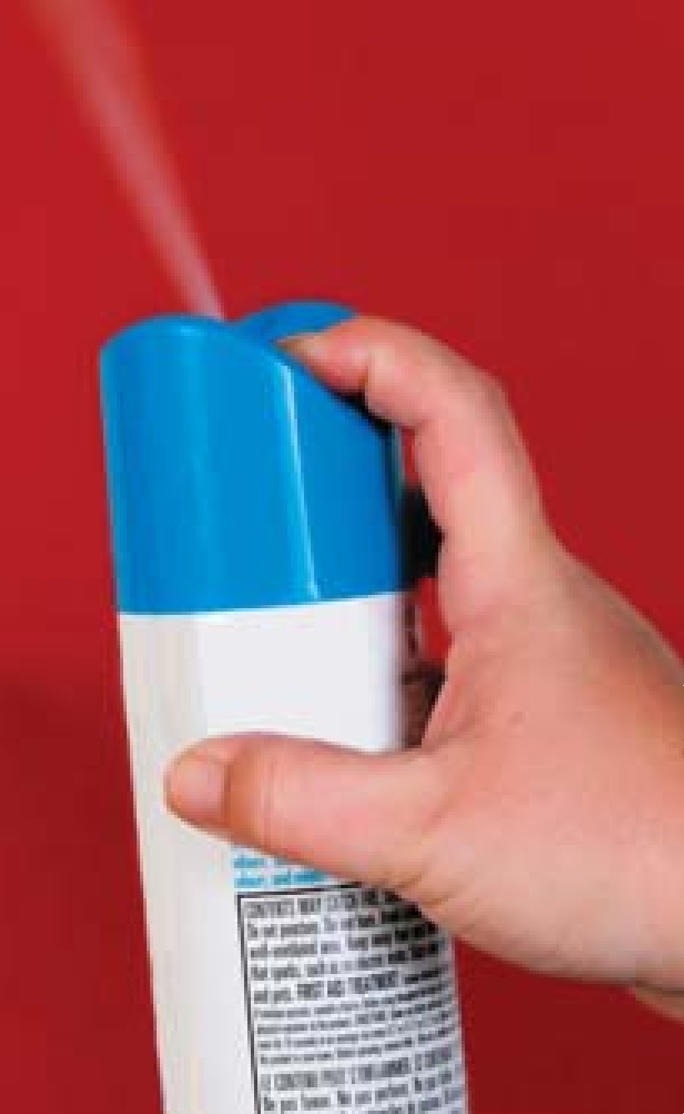# The Beat

**Published:** 2007-11

**Authors:** Erin E. Dooley

## Mercury Trackers

A first-of-its-kind whole-ecosystem study shows that atmospheric emissions of mercury from coal-fired power plants and other sources end up in fish in as little as three years. The study, published online 27 September 2007 ahead of print in the *Proceedings of the National Academy of Sciences*, traced mercury’s movement through a test watershed, yielding critical information for better understanding how the element distributes through ecosystems. The paper’s authors predict that reductions in mercury emissions could translate into reduced fish methylmercury loads within a decade.

**Figure:**
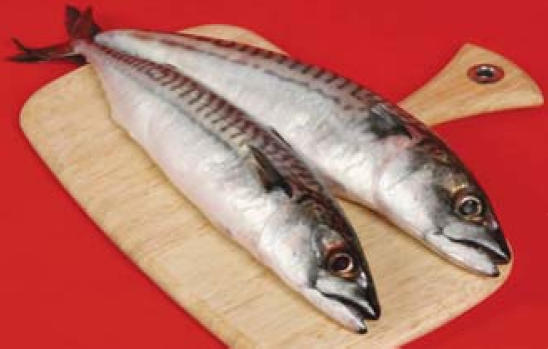


## Social Environment and Asthma

A paper in the 1 October 2007 edition of the *American Journal of Respiratory and Critical Care Medicine* supports a two-way link between asthma and a child’s social environment, as evaluated using a standard instrument. Social environment was defined in terms of family support (the degree to which parents understand, value, and care about children) and neighborhood factors including crime and violence. Less family support correlated with children having higher levels of IgE, eosinophils, and IL-4, inflammation markers associated with poorer asthma outcomes. Neighborhood problems were linked with behavioral influences on asthma, such as early initiation of smoking, exposure to secondhand smoke, and poor adherence to asthma medications.

## Melnick Receives Rall Award

The American Public Health Association has awarded its 2007 David P. Rall Award for Advocacy in Public Health to Ronald Melnick, senior toxicologist and director of special programs in the NIEHS National Toxicology Program. In his 27 years at the institute, Melnick has engaged in a wide range of activities that have advanced scientific knowledge of the adverse health effects of chemical exposures and influenced public health policy making in this area. Known as an ardent supporter of more protective chemical exposure standards based on science-based evidence, his research on such chemicals as butadiene, isoprene, glycol esters, and drinking water disinfection by-products has furthered our understanding of their toxicity potential to populations.

**Figure:**
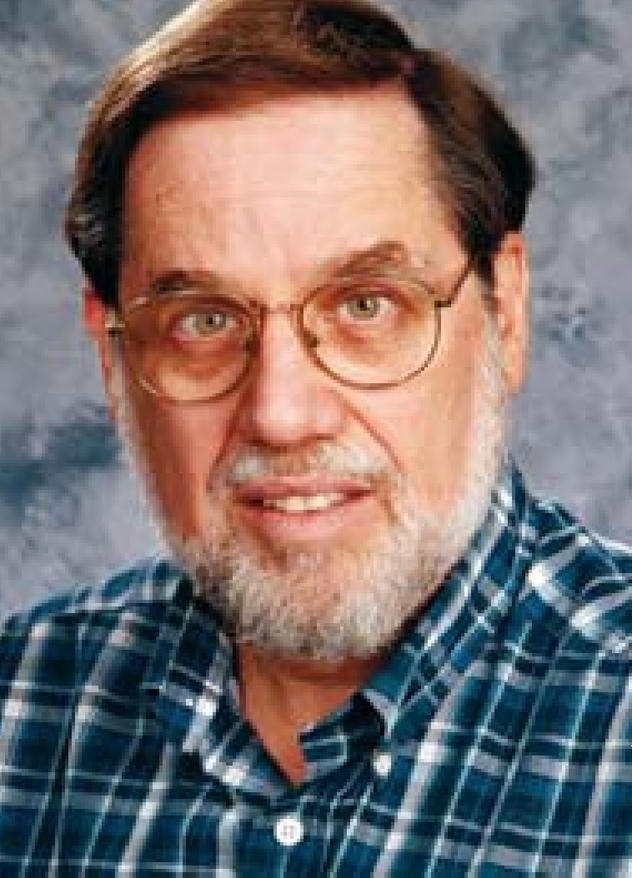


## Spice for LIfe?

Each year *Escherichia coli* causes 210 million cases of diarrhea, the leading cause of infant death in developing countries. Taiwanese researchers now report in the 3 October 2007 issue of the *Journal of Agricultural and Food Chemistry* that compound 31, a derivative of the ginger constituent zingerone, blocks *E. coli* heat-labile enterotoxin from binding to cell surfaces. Ginger has long been used as a remedy for digestive problems, and the authors propose that compound 31 could someday be used to formulate cheap, widely obtainable diarrhea medicines. First, though, dosage and possible side effects in infants need to be determined.

**Figure:**
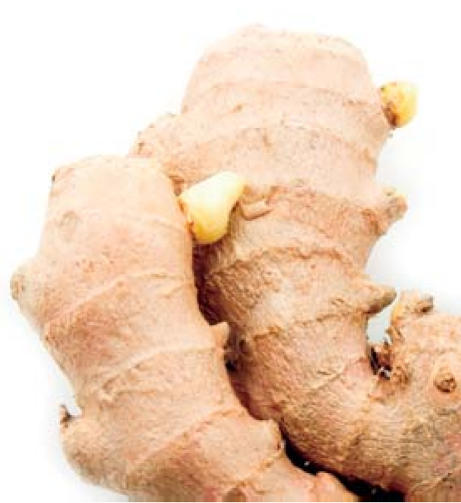


## New Bill for Environmental Health

Bipartisan legislation introduced in September 2007 by Speaker of the House Nancy Pelosi (D–CA) could boost funding for state participation in the CDC’s National Environmental Public Health Tracking Program. The Coordinated Environmental Public Health Network Act of 2007 would also expand biomonitoring capabilities and data collection at the federal and state levels; establish a national service to oversee response efforts to unusual illness incidences or environmental hazards; and mandate the biennial publication of a report describing environmental and other factors with a potential impact on the nation’s health. The bill is currently in committee in both the House and Senate.

## A Whiff of Phthalates

U.S. sales of air fresheners have grown by 50% since 2003, with an estimated 75% of households using these products, according to the Natural Resources Defense Council (NRDC), which released a September 2007 report assessing the phthalate content of 14 different air freshener brands. Twelve of the brands tested contained phthalates, but this ingredient was listed nowhere on the label because federal agencies currently apply no such regulation to these products. Following the release of the report, national retailer Walgreens—which marketed two of the products heaviest in phthalates—pulled its air fresheners from its shelves, meanwhile agreeing to perform independent safety tests and make phthalate-free air fresheners available to its customers.

**Figure:**